# Changes in Physical and Psychological States with Respect to the Gender of Outpatients Receiving Rehabilitation at Geriatric Health Services Facilities during the COVID-19 State of Emergency

**DOI:** 10.3390/diseases9030051

**Published:** 2021-07-03

**Authors:** Kazuhiro P. Izawa, Masataka Oyama, Keisuke Okamoto

**Affiliations:** 1 Department of Public Health, Graduate School of Health Sciences, Kobe University, 10-2 Tomogaoka 7-chome, Suma-ku, Kobe 654-0142, Japan; 2 Cardiovascular Stroke Renal Project (CRP), Kobe 654-0142, Japan; 3 Geriatric Health Services Facility Elder Village, 882 Fukutani, Hasetani-cho, Nishi-ku, Kobe-shi, Hyogo 651-2233, Japan; masataka.oyama@asunaro-grp.jp (M.O.); keisuke.okamoto@asunaro-grp.jp (K.O.)

**Keywords:** geriatric health services facilities, gender, rehabilitation, physical, psychological, Coronavirus disease 2019, pandemic

## Abstract

This study was a sub-analysis of 20 consecutive elderly participants who underwent outpatient rehabilitation at a geriatric health services facility from January 2020 to the end of May 2020, based on our previous report. This study aimed to evaluate the longitudinal changes in their physical and psychological states with respect to gender in rehabilitation outpatients between the pre-nationwide (T1) and post-nationwide state of emergency (T2) caused by the Coronavirus disease 2019 (COVID-19). Gait speed (GS), timed up and go (TUG), handgrip strength (HG), and maximum phonation time (MPT) were measured as indices of physical status. The Japanese version of the Apathy Scale and five-level EuroQoL five-dimensional questionnaire (EQ-5D-5L) were used to assess the psychological state. Both states were measured in the male and female groups at T1 and T2 and then were compared. The final analysis was comprised of 13 outpatients. In males, the physical (GS, *p* = 0.463; TUG, *p* = 0.600; HG, *p* = 0.753; and MPT, *p* = 0.249) and psychological (Apathy Scale, *p* = 0.891 and EQ-5D-5L, *p* = 0.249) states did not change significantly between T1 and T2. In the females, the physical (GS, *p* = 0.600; TUG, *p* = 0.735; HG, *p* = 1.000; and MPT, *p* = 0.310) and psychological (Apathy Scale, *p* = 0.588 and EQ-5D-5L, *p* = 0.176) states also did not show significant change between T1 and T2. In both sexes, the continuance of outpatient rehabilitation might be recommended as one activity that can maintain physical and psychological states during a COVID-19-related state of emergency.

## 1. Introduction

The World Health Organization declared Coronavirus disease 2019 (COVID-19) a pandemic on March 11, 2020 [1]. In Japan, the government declared an unprecedented first state of emergency on April 7, 2020, and terminated it on May 25, 2020 [2]. These policies helped to inhibit the outbreak of infection [3], but health problems can be a concern when restrictions are imposed on activity [3,4].

Previously, the physical activity of older adults was reported to have dropped significantly as a result of the COVID-19 epidemic [4,5]. Furthermore, another report found that after accounting for pre-COVID-19 trends, loneliness and lowered physical activity experienced during the pandemic were risk factors for worsening mental health [6] and that further loss of physical activity can have serious consequences. However, our previous study found that by taking effective measures against infection during a state of emergency, geriatric health services facilities in Japan have continued to perform outpatient rehabilitation. As a result, the physical and psychological states of the outpatients did not significantly decrease as determined before and after the nationwide state of emergency [7]. It is well known that gender-related differences in physical and psychological states exist [8,9,10]. So far, several gender-related reports have been published. However, it is still not clear whether the physical and psychological states of outpatients undergoing rehabilitation at a geriatric health services facility differed by gender from before to after the nationwide state of emergency. Therefore, we thought that the physical and psychological states of both males and females undergoing outpatient rehabilitation in geriatric health services facilities would not decrease from before to after the nationwide state of emergency.

The purpose of the present study was to determine whether changes occurred in the physical and psychological states with respect to the gender of outpatients undergoing rehabilitation at a geriatric health services facility as assessed before and after the nationwide state of emergency caused by the COVID-19 pandemic in Japan.

## 2. Materials and Methods

### 2.1. Participants

This study was a sub-analysis of a longitudinal study of 20 consecutive elderly participants who underwent outpatient rehabilitation at the Elder Village geriatric health services facility in Kobe, Japan, from January 2020 to the end of May 2020, based on our previous report [7]. Outpatients who did not provide informed consent, could not walk unaided, and who could not attend outpatient rehabilitation during the state of emergency due to their preference and/or personal convenience of the participant or their family were excluded from this study. Infection prevention measures including mask-wearing, handwashing, disinfection of equipment, and other measures were strictly enforced for the participants and staff [7]. The study was conducted according to the guidelines of the Declaration of Helsinki and was approved by the Institutional Review Board of the ASUNAROKAI medical corporation (Approve date: 9 April 2021). Informed consent was obtained from each patient.

Outpatients attended rehabilitation at the geriatric health services facility once, twice, or three times per week for 20 min/session during the state of emergency [11]. Each session comprised a warm-up period, upper and lower limb and body stretches, resistance training, aerobic exercise, and cooldown period [11]. Exercise intensity was such that heart rate was maintained at a perceived exertion rating of 11–13 on the Borg scale as reported previously [7,11]. Data on the characteristics of the participants were from their medical records; age, sex, body mass index (BMI), long-term care insurance level, living alone, diagnosis, and medications were evaluated.

### 2.2. Measurement of Physical Status

Gait speed [11], timed up and go test [12], handgrip strength [11,13], and maximum phonation time [14] were assessed as indices of physical status, as in our previous study [7].

### 2.3. Measurement of Psychological States

The Japanese versions of the Apathy Scale [15,16] and the five-level EuroQoL five-dimensional questionnaire (EQ-5D-5L) [17,18,19] were used to assess the outpatients’ psychological state. Apathy is defined as the absence of or lack of feeling, emotion, interest, or concern [15,16]. The Apathy Scale includes 14 questions on spontaneity, initiation, emotionality, activity level, and interest in hobbies. The participants self-assessed themselves using this scale. Each answer was scored from a grade of 0 to 3, with the total score being used for the analysis [15,16]. For Japanese people, a score of 16 points or more has been proposed to indicate a decrease in motivation [15,16].

The EQ-5D-5L is a self-reported questionnaire with which patients self-evaluate their current state of health [17,18,19]. The EQ-5D-5L evaluates five items: mobility, self-care, usual activities, pain/discomfort, and anxiety/depression, with each item having five levels of description [17,18,19]. The EQ-5D-5L QoL score, which ranges from 0 (death) to 1 (full health), is calculated by a value set determined beforehand that reflects the preferences in the general population. In addition, exercise time per week during outpatient rehabilitation was evaluated in both groups. Physical and occupational therapists assessed the results of the Apathy Scale and EQ-5D-5L at two-time points: before (January 2020; T1) and after (end of May 2020; T2) the nationwide state of emergency.

### 2.4. Statistical Analysis

Participants’ characteristics and their results are shown as numbers and as the mean ± standard deviation for the continuous variables. After collecting the data, we separated the physical and psychological assessment results into two groups by gender. As this study’s sample size was quite small, we analyzed longitudinal changes of the physical and psychological states separately in the male and female groups from T1 to T2 using the Wilcoxon signed-rank test. The overall level of statistical significance was set at 0.05. Statistical analyses were conducted with IBM SPSS Statistics 26 (IBM SPSS, Tokyo, Japan).

## 3. Results

### 3.1. Participants’ Characteristics

Among the 20 patients, five were excluded because they could not attend outpatient rehabilitation due to their preference and/or their personal convenience or that of their family, and two patients could not walk unaided. Thus, 13 patients were included in the final analysis. Of them, the male group was comprised of six patients (age, 82.3 ± 8.4 years; BMI, 23.9 ± 1.5 kg/m2; long-term care insurance support level 2, 2 of 6; care level 1, 2 of 6; care level 2, 2 of 6; and living alone, 2 of 6). Patient diagnoses included orthopedic disease in three, cardiovascular disease in two, and cerebrovascular disease in one patient. Two patients took an angiotensin II receptor blocker, three took a β-blocker, two a calcium antagonist, one a diuretic, three a statin, and two took analgesics. Exercise time per week during outpatient rehabilitation was 46.7 ± 20.6 min.

In contrast, the female group comprised seven patients (age, 84.6 ± 8.9 years; BMI, 25.2 ± 7.5 kg/m2; long-term care insurance support level 2, 7 of 7; and living alone, 1, 7 of 7). Patient diagnoses included orthopedic disease in five and internal disease in two patients. One patient took an angiotensin II receptor blocker, one a β-blocker, four a calcium antagonist, two a diuretic, two a statin, and three took analgesics. Exercise time per week during outpatient rehabilitation was 37.1 ± 7.5 min.

### 3.2. Physical and Psychological Status in the Male Group

The physical and psychological status of the male participants showed no significant change between T1 and T2 (Table 1).

### 3.3. Physical and Psychological Status in the Female Group 

Similar to the findings in the male participants, the physical and psychological status of the female participants also showed no significant change between T1 and T2 (Table 2).

## 4. Discussion

This is the first study, to the best of our knowledge, to investigate changes in physical and physiological states by gender of outpatients participating in rehabilitation at a geriatric health services facility from before to after the state of emergency caused by the COVID-19 epidemic in Japan. In the present study, we lost about 25% of candidates in the survey for “convenience” reasons. This may also be a direct effect of the pandemic. 

Previously, Yamada et al. [4] reported the use of an online survey completed by 1600 community-dwelling older adults (74.0 ± 5.6 years, 50% women, prevalence of frailty, 23%) with frailty status assessed by the Kihon Checklist, and other demographics details and questions asked regarding physical activity at two time points: January and April 2020. As a result, they found a significant decrease in total physical activity time in April 2020 when compared to January 2020. Sasaki et al. [5] also reported, in their cross-sectional study, that a self-administered questionnaire given to 999 community-dwelling residents aged 65–90 years was used to gather data on socioeconomic status, social participation, and physical activity in August 2020. Their results suggested that physical activity in both sexes decreased by approximately 5–10% after the onset of COVID-19-related social distancing restrictions.

In the present study, we showed that the physical and psychological states of both males and females did not change significantly between T1 and T2 (Table 1 and Table 2), did not decrease, and could be maintained during this period. These results suggested a tendency unlike that in other preliminary research [4,5] and that the policies enacted in Japan during the state of emergency did not appear to affect either state in the outpatients undergoing rehabilitation.

However, Sasaki et al. [5] also reported that in terms of gender-related physical activity, men with low socioeconomic status were less physically active but that the odds of maintaining physical activity during the restrictions were higher in women engaged in social participation. In addition, they suggested that older adults with higher levels of socioeconomic status and social participation prior to the COVID-19 pandemic may have been able to maintain physical activity during the COVID-19 pandemic. Our gender-based results tend to support those of Sasaki et al. [5]. In fact, mean exercise times per week during outpatient rehabilitation in the males and females of the present study were 46.7 ± 20.6 and 37.1 ± 7.5 min, respectively.

One reason may be that the geriatric health services facility continued outpatient rehabilitation according to their policy even during the state of emergency and took adequate safety precautions against COVID-19 infection to help protect the participants. Thus, outpatient rehabilitation as a means of social participation prior to the COVID-19 pandemic might have helped outpatients of both genders maintain their physical status during the pandemic.

During the COVID-19 pandemic, mental health data were collected online by Creese et al. [6] from a preexisting group of 3281 participants older than 50 who were taking part in a longitudinal study of aging between 2015 and 2019. They analyzed trajectories of depression and anxiety between 2015 and 2020 in terms of loneliness and physical activity levels. After accounting for pre-COVID-19 trends, they suggested that experiencing loneliness and decreased physical activity during the pandemic were risk factors for worsening mental health [6] and that further loss of physical activity could have serious consequences.

Therefore, if we continue outpatient rehabilitation during the policies enacted during the state of emergency by the Japanese government, the physical and psychological status of the outpatients might not decrease in either gender. In other words, the continuance of outpatient rehabilitation during the study period may have contributed to the maintenance of their physical and psychological states.

The present study has several limitations. This study was a sub-analysis of a previous study, only one geriatric health services facility was investigated, the sample size was quite small, the longitudinal evaluation was a short amount of time, and there were several differences in restrictive national policies compare to other countries. Furthermore, there were no control groups, so we could not compare our results with those of patients not participating in outpatient rehabilitation during the state of emergency. Moreover, although we did evaluate longitudinal changes, we could not evaluate cross-sectional gender-related differences in physical and psychological states at T1 and T2. In fact, the female group, which was older and had a higher BMI than the male group, spent less time in rehabilitation. In a future study, we need to determine whether the females underwent less rehabilitation than the males because they were older.

Additionally, we investigated exercise time during outpatient rehabilitation per week but did not evaluate other activities of the outpatients, such as recreation or sedentary behavior at the geriatric health services facility by gender. Likewise, previous studies [20,21] suggested that the drop in performance of those with a pre-covid disability was smaller than the drop in performance for those more active before. However, we did not investigate this, so we need to do more research in future trials. Furthermore, we could not assume that all patients were mentally healthy in the present study. This was a big limitation and very important for the analysis and discussions of the results. Finally, as there is no long-term data on gender-related differences in the outcomes of these patients, the generalizability of the present results may be limited.

## 5. Conclusions

The present sub-analysis investigation of both males and females who participated in outpatient rehabilitation at a geriatric health services facility showed that neither gender appeared to have experienced a decrease in their physical and psychological states during the state of emergency brought on by the COVID-19 pandemic in Japan.

## Figures and Tables

**Table 1:** Physical and Psychological Status in the Male Group.

Status	T1	T2	*p*-Value
**Physical**			
Gait speed (m/sec)	1.08 ± 0.36	1.06 ± 0.32	0.463
Timed up and go (sec)	11.24 ± 4.09	10.81 ± 2.68	0.6
Handgrip strength (kgf)	22.91 ± 6.14	23.42 ± 8.19	0.753
Maximum phonation time (sec)	11.44 ± 3.99	12.56 ± 3.25	0.249
**Psychosocial**			
Apathy score	18.30 ± 5.82	18.30 ± 5.43	0.891
EQ-5D-5L QoL score	0.69 ± 0.13	0.79 ± 0.21	0.249

Data are presented as mean ± standard deviation. EQ-5D-5L, five-level version of the EuroQoL five-dimensional questionnaire; QoL, quality of life; T1, January 2020; T2, end of May 2020.

**Table 2:** Physical and Psychological Status in the Female Group.

Status	T1	T2	*p*-Value
**Physical**			
Gait speed (m/sec)	0.79 ± 0.25	0.80 ± 0.33	0.6
Timed up and go (sec)	16.53 ± 6.83	17.00 ± 7.71	0.735
Handgrip strength (kgf)	16.43 ± 3.81	16.52 ± 4.32	1
Maximum phonation time (sec)	15.49 ± 4.96	14.84 ± 3.16	0.31
**Psychosocial**			
Apathy score	15.60 ± 7.18	14.40 ± 9.95	0.588
EQ-5D-5L QoL score	0.77 ± 0.09	0.83 ± 0.87	0.176

Data are presented as mean ± standard deviation. EQ-5D-5L, five-level version of the EuroQoL five-dimensional questionnaire; QoL, quality of life; T1, January 2020; T2, end of May 2020.

## Data Availability

No new data were created or analyzed in this study. Data sharing is not applicable to this article.

## References

[1] World Health Organization Director-General’s Opening Remarks at the Media Briefing on COVID-19–11 March 2020. https://www.who.int/dg/speeches/detail/who-director-general-s-opening-remarks-at-the-media-briefing-on-covid-19---11-march-2020.

[2] [COVID-19] Guidelines for Lifting the State of Emergency. http://japan.kantei.go.jp/ongoingtopics/_00025.html.

[3] Hsiang S., Allen D., Annan-Phan S., Bell K., Bolliger I., Chong T., Druckenmiller H., Huang L.Y., Hultgren A., Krasovich E. (2020). The effect of large-scale anti-contagion policies on the COVID-19 pandemic. Nature.

[4] Yamada M., Kimura Y., Ishiyama D., Otobe Y., Suzuki M., Koyama S., Kikuchi T., Kusumi H., Arai H. (2020). Effect of the COVID-19 epidemic on physical activity in community-dwelling older adults in Japan: A cross-sectional online survey. J. Nutr. Health Aging.

[5] Sasaki S., Sato A., Tanabe Y., Matsuoka S., Adachi A., Kayano T., Yamazaki H., Matsuno Y., Miyake A., Watanabe T. (2021). Associations between socioeconomic status, social participation, and physical activity in older people during the COVID-19 pandemic: A cross-sectional study in a northern Japanese city. Int. J. Environ. Res. Public Health.

[6] Creese B., Khan Z., Henley W., O’Dwyer S., Corbett A., Da Silva M.V., Mills K., Wright N., Testad I., Aarsland D. (2020). Loneliness, physical activity, and mental health during COVID-19: A longitudinal analysis of depression and anxiety in adults over the age of 50 between 2015 and 2020. Int. Psychogeriatr..

[7] Izawa K.P., Oyama M., Okamoto K. (2020). Did the physical and psychological states of outpatients receiving rehabilitation at a ger-iatric health services facility decline during the state of emergency caused by the COVID-19 pandemic?. Diseases.

[8] Izawa K.P., Oka K., Watanabe S., Yokoyama H., Hiraki K., Morio Y., Kasahara Y., Omiya K. (2008). Gender-related differences in clinical characteristics and physiological and psychosocial outcomes of Japanese patients at entry into phase II cardiac rehabilitation. J. Rehabil. Med..

[9] Izawa K.P., Watanabe S., Hirano Y., Matsushima S., Suzuki T., Oka K., Kida K., Suzuki K., Osada N., Omiya K. (2015). Gender-related differences in maximum gait speed and daily physical activity in elderly hospitalized cardiac inpatients: A preliminary study. Medicine.

[10] Izawa K.P., Shibata A., Ishii K., Miyawaki R., Oka K. (2017). Associations of low-intensity light physical activity with physical performance in community-dwelling elderly Japanese: A cross-sectional study. PLoS ONE.

[11] Izawa K.P., Kasahara Y., Hiraki K., Hirano Y., Oka K., Watanabe S. (2019). Longitudinal changes of handgrip, knee extensor muscle strength, and the disability of the arm, shoulder and hand score in cardiac patients during phase II cardiac rehabilitation. Diseases.

[12] Ogawa M., Izawa K.P., Satomi-Kobayashi S., Kitamura A., Tsuboi Y., Komaki K., Ono R., Sakai Y., Tanaka H., Okita Y. (2018). Preoperative exercise capacity is associated with the prevalence of postoperative delirium in elective cardiac surgery. Aging Clin. Exp. Res..

[13] Izawa K.P., Watanabe S., Oka K. (2015). Relationship of thresholds of physical performance to nutritional status in older hospitalized male cardiac patients. Geriatr. Gerontol. Int..

[14] Izawa K.P., Watanabe S., Tochimoto S., Hiraki K., Morio Y., Kasahara Y., Watanabe Y., Tsukamoto T., Osada N., Omiya K. (2012). Relation between maximum phonation time and exercise capacity in chronic heart failure patients. Eur. J. Phys. Rehabil. Med..

[15] Okada K., Kobayashi S., Yamagata S., Takahashi K., Yamaguchi S. (1997). Poststroke apathy and regional cerebral blood flow. Stroke.

[16] Yamagata S., Yamaguchi S., Kobayashi S. (2004). Impaired novelty processing in apathy after subcortical stroke. Stroke.

[17] Shiroiwa T., Fukuda T., Ikeda S., Igarashi A., Noto S., Saito S., Shimozuma K. (2016). Japanese population norms for preference-based measures: EQ-5D-3L, EQ-5D-5L, and SF-6D. Qual. Life Res..

[18] Shiroiwa T., Ikeda S., Noto S., Igarashi A., Fukuda T., Saito S., Shimozuma K. (2016). Comparison of value set based on DCE and/or TTO data: Scoring for EQ-5D-5L health states in Japan. Value Health.

[19] Herdman M., Gudex C., Lloyd A., Janssen M.F., Kind P., Parkin D., Bonsel G., Badia X. (2011). Development and preliminary testing of the new five-level version of EQ-5D (EQ-5D-5L). Qual. Life Res..

[20] McCarthy H., Potts H.W.W., Fisher A. (2021). Physical Activity Behavior Before, During, and After COVID-19 Restrictions: Longitudinal Smartphone-Tracking Study of Adults in the United Kingdom. J. Med. Internet Res..

[21] To Q.G., Duncan M.J., Van Itallie A., Vandelanotte C. (2021). Impact of COVID-19 on Physical Activity Among 10,000 Steps Members and Engagement With the Program in Australia: Prospective Study. J. Med. Internet Res..

